# Molecular Deconvolution of Circulating “Other Cells”: A Reliable Predictive Marker of Therapy Response and Survival in Neuroendocrine Tumor Patients Receiving ^177^Lu‐DOTATATE PRRT

**DOI:** 10.1002/cam4.71444

**Published:** 2026-01-07

**Authors:** Mahesh K. Padwal, Rahul V. Parghane, Sandip Basu, Bhakti Basu

**Affiliations:** ^1^ Molecular Biology Division Bhabha Atomic Research Centre Mumbai India; ^2^ Homi Bhabha National Institute Mumbai India; ^3^ Radiation Medicine Centre Bhabha Atomic Research Centre, Tata Memorial Centre Annexe Mumbai India

**Keywords:** ^177^Lu‐DOTATATE PRRT, disease control rate, neuroendocrine tumors, other cell proportions, prognosis

## Abstract

**Purpose:**

To assess the prognostic impact of pre‐treatment circulating other cells on treatment response and survival in neuroendocrine tumor (NET) patients receiving Peptide Receptor Radionuclide Therapy (PRRT) with ^177^Lu‐DOTATATE.

**Methods:**

Healthy donors (HDs, *n* = 81) and NET patients (*n* = 137) were analyzed. Baseline circulating other cells proportions (OC%) were deconvoluted from RNA‐Seq profiles using the Kassandra‐B algorithm. Associations of OC% with demographic and clinicopathologic characteristics, tumor response to PRRT, and survival outcomes were evaluated.

**Results:**

NET patients had a significantly higher OC% compared to HDs (median = 0.26 vs. 0.18, *p* = 0.0018). A positive correlation was observed between OC% and the tumor grade (G3 tumors: median = 0.47, G1 tumors: median = 0.22, *p* = 0.029) or disease burden (high: median = 0.32, low: median = 0.23, *p* = 0.016). The OC% was negatively associated with ^68^Ga‐DOTATATE avidity (high: median = 0.22, low: median = 0.32, *p* = 0.0026). Patients with progressive disease had high OC% (median = 0.41, IQR = 0.25–0.56), significantly higher than those with stable disease (median = 0.24, IQR = 0.12–0.36, *p* = 0.0024) or complete/partial response (median = 0.22, IQR = 0.08–0.32, *p* = 0.0067). NET patients in the OC^Low^ group (OC% ≤ 0.18%) had > 90% disease control rate on PRRT, independent of tumor characteristics. Patients in the OC^High^ group (OC% > 0.18%) had a significantly higher risk of early disease progression [HR = 3.2, 95% CI = 1.32–7.75, *p* = 0.01]. After adjusting for demographic and tumor characteristics, the association of OC% with progression‐free survival retained marginal significance [HR = 2.08, 95% CI = 0.78–5.54, *p* = 0.14], in a multivariate model. Integration of OC% with established risk variables identified a specific subgroup of NET patients at an increased risk of PRRT failure and poor prognosis.

**Conclusions:**

Pre‐treatment OC%, in conjunction with critical risk factors, is predictive of treatment response and survival in NET patients receiving ^177^Lu‐DOTATATE PRRT. This RNA‐Seq‐based test may evolve as a dependable adjunct for clinicians in the risk stratification of NET patients, in the PRRT setting.

**Abbreviations**
NET: neuroendocrine tumor
OC: other cells
PRRT: peptide receptor radionuclide therapy

## 1 Introduction

Neuroendocrine tumors (NETs) represent rare malignancies of neuroendocrine cells diffusely spread across the body, with the majority originating in the gastrointestinal tract, pancreas, and lungs [1, 2, 3]. The incidence of NETs has rapidly increased over recent years [4]. These indolent tumors often present at an advanced metastatic stage resulting in heterogeneous clinical manifestations [5]. The diverse origin and biology of NETs further confound the clinical outcome [6]. Currently, the treatment options for NETs comprise targeted therapies with sunitinib (tyrosine kinase inhibitor) and everolimus (mTOR inhibitor), somatostatin analogs, peptide receptor radionuclide therapy (PRRT), or chemotherapy [5, 7, 8]. In gastroenteropancreatic NET (GEP‐NET) patients, prognosis is independently influenced by tumor grade, disease burden, distant metastasis, ^68^Ga‐DOTATATE avidity, baseline neutrophil‐to‐lymphocyte ratio or monocyte‐to‐lymphocyte ratio, and the activity of systemic cancer hallmarks such as heme metabolism or IL2/STAT5 signaling [9, 10].

The circulating tumor cells (CTCs) are considered the “seeds” of cancer metastasis that establish new tumors at distant locations [11]. Despite significant advances in treatment protocols, metastasis remains one of the most critical determinants of clinical outcome and prognosis in cancer patients [12]. The presence of CTCs in venous blood has been associated with cancer metastasis, early recurrence and shorter survival across several cancers [11]. Thus, CTCs in blood are considered the harbingers of advanced‐stage disease and poor prognosis [13]. The prognostic significance of CTCs has been reported in several solid tumors of breast, prostate, colorectal and other origins [14, 15]. In NETs, Khan et al. reported worse progression‐free survival (PFS) and overall survival (OS) in metastatic NET patients with ≥ 1 CTCs in 7.5 mL blood [16]. Mandair et al. determined 1 and 2 CTCs/7.5 mL blood as the optimal threshold to predict PFS as well as OS in metastatic pancreatic and midgut NETs, respectively [17]. In the Asian population, tumor grade and baseline CTC counts had an independent prognostic impact on PFS as well as OS [18]. However, the detection of CTCs remains a challenge due to their scarcity (0.1–10 CTCs/mL of whole blood) [19]. Apart from CTCs, circulating stromal cells [20, 21], endothelial cells [22, 23], and epithelial cells [24] are emerging blood‐based biomarkers for screening invasive solid tumors and predicting clinical outcomes. However, except for CTCs, there is a gap in knowledge regarding the prognostic potential of these cells in NETs.

Conventionally, flow cytometry has been a *workhorse* for phenotyping cell subsets in clinical samples, especially in the context of cancers [25]. Studies on circulating cells require very sophisticated and expensive technologies with size‐based enrichment (CellSieve microfiltration) and/or marker‐based enrichment. However, the high costs of detection methods limit their translation from research to clinics. Advancements in next‐generation sequencing technology and computational tools have enabled the development of deconvolution algorithms for the estimation of unique cell type proportions from the bulk RNA‐Seq profiles of the tissue or blood samples [26, 27]. The Kassandra algorithm [27] accurately estimates 40 phenotypically characterized immune cell types and “other cells” in blood samples. Essentially, “other cells” comprise all circulating cells inclusive of uncharacterized immune cells, CTCs, stromal cells, endothelial cells, or epithelial cells [27]. In this study, pre‐treatment “other cells” proportions (OC%) were estimated from the peripheral blood RNA‐Seq profiles and their prognostic impact was evaluated in NET patients treated with ^177^Lu‐DOTATATE PRRT.

## 2 Materials and Methods

### 2.1 Study Participants

This study was conducted at a single tertiary‐care institution and involved asymptomatic healthy donors (*n* = 81) and NET patients (*n* = 137) with advanced, unresectable, or metastatic tumors. Baseline demographic data (age and sex), tumor characteristics (primary site, tumor grade, disease burden, presence of liver or bone metastases, [68Ga]‐DOTA‐D‐Phe‐Tyr3‐octreotate (^68^Ga‐DOTATATE) avidity and [18F]‐fluorodeoxyglucose (^18^F‐FDG) avidity), response to PRRT and survival data were collected for NET patients (Table 1 and Appendix S1). Clinical data were independently reviewed and verified by two clinicians. Healthy donors (HDs) without any documented co‐morbid conditions were enrolled in the study. HD samples were anonymized after recording demographic data. The study was reviewed and approved by the Institutional Scientific Advisory Committee (SAC) and the Institutional Ethics Committee (IEC) at the Radiation Medicine Center, Bhabha Atomic Research Center (Approval No. P18/Feb/2019). The study was carried out as per the ethical tenets of the Helsinki Declaration. All participants signed written informed consent.

**TABLE 1:** Demographic and clinicopathological features of study participants.

Healthy donors (*n* = 81)	
Median age (IQR, min–max)	45 years (36, 56)
Sex	
Female, *n* (%)	27 (33.33%)
Male, *n* (%)	54 (66.67%)

NET patients (*n* = 137)	
Median age (IQR, min–max)	50 years (43, 59)
Sex	
Female, *n* (%)	55 (40.14%)
Male, *n* (%)	82 (59.85%)
Primary site	
Pancreas, *n* (%)	71 (51.82%)
Gastric, *n* (%)	6 (4.38%)
Ileum, *n* (%)	16 (11.68%)
Duodenum, *n* (%)	10 (7.3%)
Jejunum, *n* (%)	2 (1.46%)
Rectal, *n* (%)	13 (9.49%)
Colon, *n* (%)	1 (0.73%)
Lung, *n* (%)	5 (3.65%)
CUP, *n* (%)	7 (5.11%)
Others, *n* (%)	6 (4.38%)
Tumor grade	
G1, *n* (%)	45 (32.85%)
G2, *n* (%)	83 (60.58%)
G3, *n* (%)	9 (6.57%)
Disease burden	
Low, *n* (%)	93 (67.88%)
High, *n* (%)	44 (32.12%)
Metastasis	
No, *n* (%)	26 (18.98%)
Liver, *n* (%)	87 (63.50%)
Bone, *n* (%)	24 (17.52%)
^68^Ga‐DOTATATE avidity	
Low (SUVmax ≤ 20), *n* (%)	36 (26.28%)
High (SUVmax > 20), *n* (%)	101 (73.72%)
^18^F‐FDG avidity	
Low (SUVmax ≤ 5), *n* (%)	65 (47.45%)
High (SUVmax > 5), *n* (%)	72 (52.55%)
Response to PRRT (RECIST 1.1)	
Complete response (CR), *n* (%)	1 (0.73%)
Partial response (PR), *n* (%)	31 (22.63%)
Stable disease (SD), *n* (%)	85 (62.04%)
Progressive disease (PD), *n* (%)	20 (14.6%)
Follow‐up of survival	
Median PFS, (IQR, min–max)	25 months (16, 37)
Median OS, (IQR, min–max)	26 months (18, 39)
Disease progression, *n* (%)	35 (25.55%)
Deaths, *n* (%)	17 (11.77%)

### 2.2 Diagnosis and Treatment of NET Patients

NET patients underwent radiological and histopathological evaluations as part of the institutional standard PRRT workup [8]. Tumor metabolism and heterogeneity were examined with dual‐tracer diagnostic imaging with ^68^Ga‐DOTATATE and ^18^F‐FDG PET/CT. Inclusion criteria: Krenning score ≥ 3 on ^68^Ga‐DOTATATE PET/CT, indicating advanced metastatic disease or progressive disease on other treatments. Exclusion criteria: Krenning score ≤ 2, Eastern Cooperative Oncology Group (ECOG) performance status of 3–4, Karnofsky performance status score < 30, or inadequate hematological, hepatic, or renal function. Eligible NET patients underwent [177Lu]‐DOTA‐D‐Phe‐Tyr3‐octreotate (^177^Lu‐DOTATATE) Peptide Receptor Radionuclide Therapy (PRRT). Patients received 4–6 cycles of ^177^Lu‐DOTATATE PRRT (150–200 mCi per cycle) at intervals of 10–12 weeks [8]. Tumor response to PRRT was assessed according to RECIST 1.1 criteria [28], at 3–6 months after the last cycle of PRRT. Patients achieving complete response (CR) or partial response (PR) were RECIST “responders”, while those with stable disease (SD) or progressive disease (PD) were “non‐responders” according to RECIST 1.1 criteria [28]. Progression‐free survival (PFS) and overall survival (OS) data were collected during post‐PRRT follow‐up (Table 1).

### 2.3 Other Cells Proportions

Previously reported RNA‐Seq datasets of pre‐PRRT blood samples from NET patients [9, 29] and healthy donors [9, 29] were analyzed in this study. Molecular proportions of “other” cells were estimated with the Kassandra‐Blood algorithm [27] using Kallisto [30] quantified transcript abundance values (Appendix S1). Batch effects were adjusted through TPM normalization employed by the Kassandra‐B algorithm prior to the estimation of cell proportions.

### 2.4 Statistical and Survival Analysis

Statistical analyses were conducted in R (v4.3) [31]. Continuous variables were summarized as median with interquartile range (IQR), while categorical variables were presented as absolute frequencies and percentages. Comparisons of continuous variables were performed using the Wilcoxon rank‐sum test with continuity correction. A *p* value of ≤ 0.05 was considered statistically significant. The median OC% in the healthy donor (HD) cohort was used as a cut‐off to dichotomize NETs patients into OC^Low^ and OC^High^ groups. Survival outcomes assessed in terms of progression‐free survival (PFS) and overall survival (OS) were the primary and secondary endpoints, respectively. PFS was defined as the duration (in months) from the initial PRRT administration to disease progression or last follow‐up for patients without progression. OS was defined as the time (in months) from the first PRRT cycle to death or last follow‐up for surviving patients. Disease control rate (DCR) was defined as the proportion of patients achieving complete response (CR), partial response (PR), or stable disease (SD) on RECIST 1.1 criteria. Univariate and multivariate Cox proportional hazards (COXPH) regression models were employed to estimate hazard ratios (HRs) and 95% confidence intervals (CIs). The proportional hazards assumption was assessed using the COX.ZPH function with rank‐based transformation. Kaplan–Meier survival curves were generated using the survminer package in R. Year‐wise disease control rates were calculated using Kaplan–Meier analysis, and significance was tested through a *z*‐test at each time point.

## 3 Results

### 3.1 Demographic Comparison of Other Cells Proportions in HDs and NET Patients

Digitally deconvoluted OC% in HDs and NET patients were analyzed. In the HDs (*n* = 81) and NET patients (*n* = 137) cohorts, age or sex did not significantly influence OC% (Table 2). In the HD cohort, the median OC% was 0.18 (IQR = 0.05–0.28). OC% did not show a significant difference with respect to age (≤ 50 years: median = 0.18, IQR = 0.04–0.30 vs. > 50 years: 0.15, IQR = 0.05–0.25; *p* = 0.85) or sex (Female: median = 0.2, IQR = 0.11–0.275 vs. Male: median = 0.15, IQR = 0.05–0.30; *p* = 0.577) in the HDs cohort (Table 2). In the NET patients cohort, the median OC% was 0.26 (IQR = 0.12–0.38); however, no significant differences were observed between males (median = 0.29, IQR = 0.12–0.43) and females (median = 0.24, IQR = 0.12–0.34) (*p* = 0.22) or between patients aged ≤ 50 years (median = 0.28, IQR = 0.12–0.38) and those > 50 years (median = 0.26, IQR = 0.12–0.39) (*p* = 0.76) (Table 2).

**TABLE 2:** Association of OC% with clinical characteristics.

Pre‐PRRT clinical characteristics	Median OC% (IQR)	*p*
Healthy donors		
HDs	0.18 (0.05–0.28)	—
Age: Low (≤ 50 years)	0.18 (0.04–0.30)	0.85
Age: High (> 50 years)	0.15 (0.05–0.25)	
Sex: Female	0.2 (0.11–0.275)	0.58
Sex: Male	0.15 (0.05–0.30)	
NET patients		
Sex: Female	0.24 (0.12–0.34)	0.22
Sex: Male	0.29 (0.12–0.43)	
Age: Low (≤ 50 years)	0.28 (0.12–0.38)	0.76
Age: High (> 50 years)	0.26 (0.12–0.39)	
HDs	0.18 (0.05–0.28)	**0.0018**
NETs	0.26 (0.12–0.38)	
Tumor grade: G1	0.22 (0.08–0.35)	0.554 (G1 vs. G2)
Tumor grade: G2	0.27 (0.12–0.38)	0.059 (G2 vs. G3)
Tumor grade: G3	0.47 (0.26–0.58)	**0.029 (G1 vs. G3)**
Low disease burden	0.23 (0.1–0.35)	**0.016**
High disease burden	0.32 (0.2–0.44)	
No bone metastasis	0.26 (0.12–0.38)	0.667
Bone metastasis	0.25 (0.14–0.41)	
No liver metastasis	0.27 (0.09–0.35)	0.747
Liver metastasis	0.23 (0.13–0.4)	
^68^Ga‐DOTATATE avidity (> 20)	0.22 (0.08–0.37)	**0.0026**
^68^Ga‐DOTATATE avidity (≤ 20)	0.32 (0.24–0.47)	
^18^F‐FDG avidity (> 5)	0.3 (0.16–0.41)	0.131
^18^F‐FDG avidity (≤ 5)	0.23 (0.08–0.35)	
Primary site (GI)	0.31 (0.16–0.38)	0.360 (GI vs. PAN)
Primary site (PAN)	0.29 (0.17–0.42)	0.885 (GI vs. other)
Primary site (Other)	0.23 (0.09–0.37)	0.374 (PAN vs. other)
Response: CR + PR	0.22 (0.08–0.32)	0.550 (CR + PR vs. SD)
Response: SD	0.24 (0.12–0.36)	**0.0067 (CR + PR vs. PD)**
Response: PD	0.41 (0.25–0.56)	**0.0024 (SD vs. PD)**

*Note:* Bold values indicate significant associations (*p* < 0.05).

### 3.2 Association of Pre‐Treatment OC% With Tumor Characteristics

**FIGURE 1:**
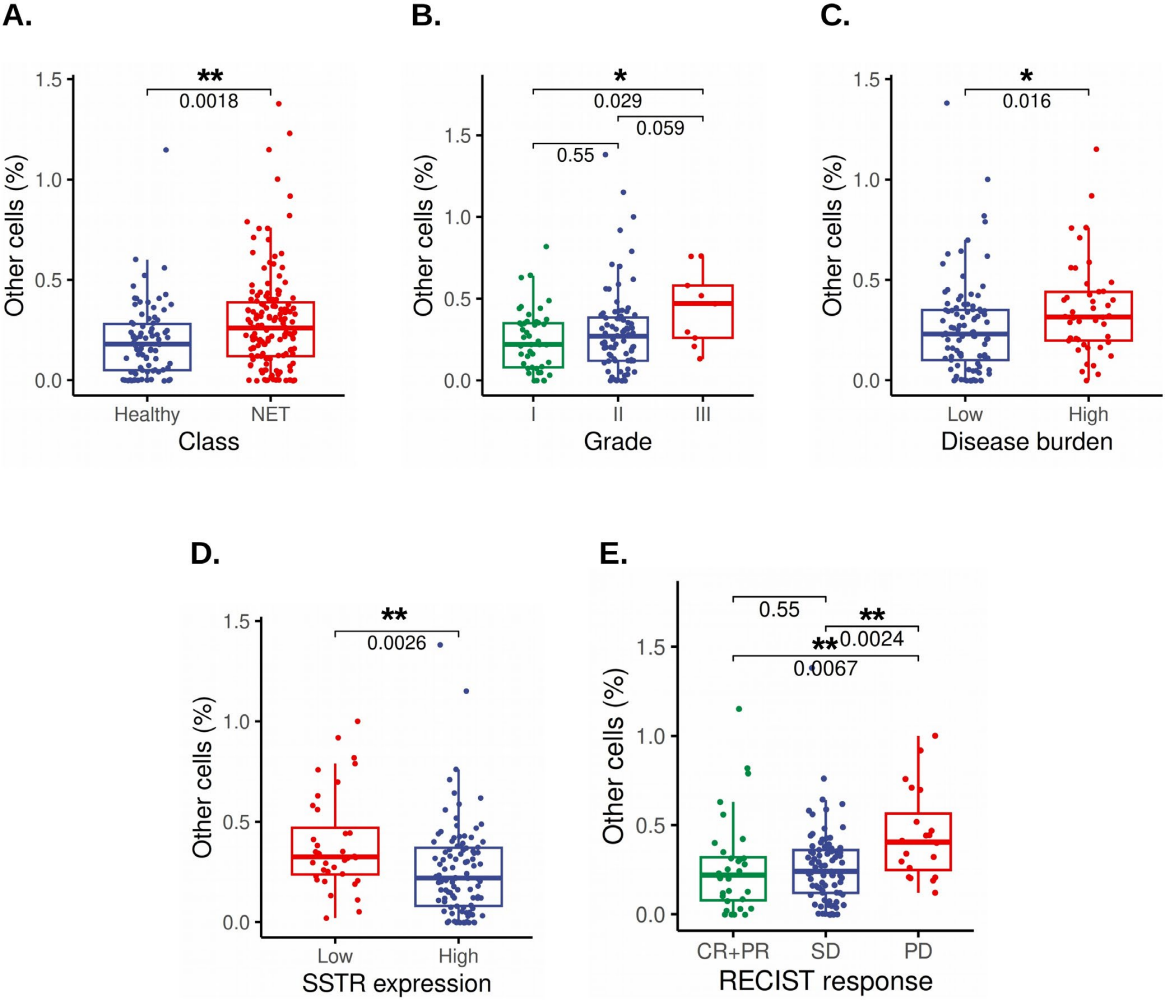
Box plots illustrating the relationship of OC% (A) in HDs versus NET patients, (B) with tumor grade, (C) with disease burden, (D) with SSTR expression, and (E) with tumor response to PRRT according to RECIST 1.1 response criteria (CR, PR, SD, PD). The *p* values are indicated at the top of each plot. In these box plots, the boxes represent the interquartile range (IQR), the horizontal lines within the boxes indicate the median, and the whiskers extend to 1.5 times the IQR. Statistical significance was assessed using the Wilcoxon rank‐sum test with continuity correction. SSTR, somatostatin receptor. *p ≤ 0.05, and **p < 0.01.

NET patients had significantly higher OC% compared to HDs (median = 0.26 vs. 0.18, *p* = 0.0018) (Figure 1A and Table 2). The association of OC% with clinicopathological characteristics in NET patients was tested (Table 2). A positive correlation was observed between the OC% and the tumor grade. Higher OC% was observed in G3 tumors (median = 0.47, IQR = 0.26–0.58) compared to G2 (median = 0.27, IQR = 0.12–0.38) or G1 (median = 0.22, IQR = 0.08–0.35). The difference was significant between G3 and G1 tumors (*p* = 0.029) and moderately significant between G3 and G2 tumors (*p* = 0.059) (Figure 1B and Table 2). NET patients with high disease burden had significantly higher OC% (median = 0.32, IQR = 0.2–0.44) compared to those with low disease burden (median = 0.23, IQR = 0.1–0.35) (*p* = 0.016) (Figure 1C and Table 2). Patients with higher ^68^Ga‐DOTATATE avidity (SUVmax > 20) had lower OC% (median = 0.22, IQR = 0.08–0.37) compared to those with lower ^68^Ga‐DOTATATE avidity (SUVmax ≤ 20) (median = 0.32, IQR = 0.24–0.47) (*p* = 0.0026) (Figure 1D and Table 2). OC% was not significantly associated with primary tumor sites (*p* > 0.3 for all comparisons), ^18^F‐FDG avidity (*p* = 0.131) or with bone or liver metastases (*p* > 0.5). However, the lower quartile values were higher in patients with liver metastases or high ^18^F‐FDG avidity compared to those without metastases or low ^18^F‐FDG avidity, respectively (Table 2).

### 3.3 Association of Pre‐PRRT OC% With Efficacy of PRRT

Higher OC% was associated with poorer response to PRRT. Patients with progressive disease (PD) had the highest median OC% (0.41, IQR = 0.25–0.56), which was significantly higher than those with stable disease (SD) (0.24, IQR = 0.12–0.36, *p* = 0.0024), or complete and partial response (CR + PR) (0.22, IQR = 0.08–0.32, *p* = 0.0067). However, the difference between CR + PR and SD was not statistically significant (*p* = 0.550) (Figure 1E and Table 2).

**FIGURE 2:**
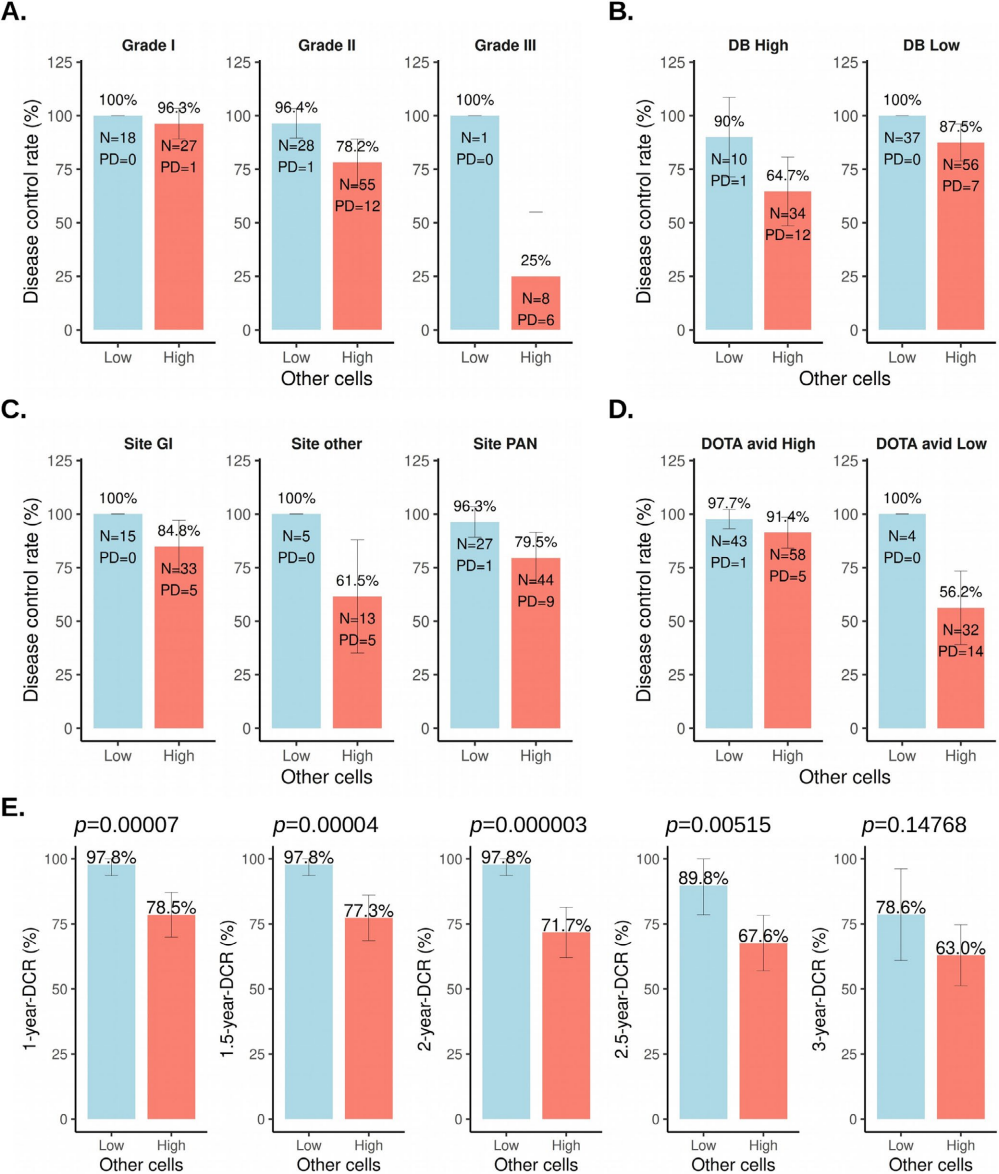
Bar plots illustrate the disease control rate (DCR = CR + PR + SD) based on (A) Tumor grade, (B) Disease burden (DB), (C) Primary site, (D) ^68^Ga‐DOTATATE (DOTA) avidity, and (E) Time from first cycle of PRRT. Error bars denote a 95% confidence interval for the response. The “Low other cells” group had OC% ≤ 0.18, and the “high other cells” group had OC% > 0.18. Tumor grades were according to the WHO 2022 classification [32]. The “DB High” group had lesion size > 7 cm and no. of lesions > 10, and the “DB Low” group had lesion size < 7 cm and no. of lesions ≤ 10. “Site GI” included NETs of the gastric, ileum, duodenum, jejunum, colon, and rectum. “Site other” included NETs of the lung, CUP, thymus, mediastinum, liver, and gall bladder. “Site PAN” included pancreatic NETs. The “DOTA avid High” group had high ^68^Ga‐DOTATATE avidity (SUVmax > 20), and the “DOTA avid Low” group had low ^68^Ga‐DOTATATE avidity (SUVmax ≤ 20). *p* values are indicated at the top in the panel (E).

Further, the association of OC% with the disease control rate (DCR) was evaluated. The patients were stratified into OC^High^ and OC^Low^ groups using the median cut‐off of 0.18%. With respect to tumor grades, patients in the OC^Low^ group had a higher DCR. In patients with G1 tumors, the DCR was 100% in the OC^Low^ group compared to 96.3% in the OC^High^ group (Figure 2A). Similarly, in patients with G2 tumors, the DCR was 96.4% in the OC^Low^ group versus 78.2% in the OC^High^ group (Figure 2A). In G3 tumor patients, the DCR was 100% in the OC^Low^ group, while disease was controlled in only 25% of patients in the OC^High^ group (Figure 2A). Of note, a single G3 patient in the OC^Low^ group had SD following PRRT, as per RECIST 1.1 criteria.

NET patients with high disease burden had DCRs of 90% and 64.7% in the OC^Low^ and OC^High^ groups, respectively (Figure 2B). DCRs improved to 100% and 87.5%, respectively, in OC^Low^ and OC^High^ groups of NET patients with low disease burden (Figure 2B). The DCR was 100% in NETs of gastrointestinal or other primary sites (including lung NETs, carcinoma of unknown primary CUP‐NETs, and rarer sites such as thymus, mediastinum, liver and gall bladder) compared to 96.3% in pancreatic NETs, in the OC^Low^ group (Figure 2C). In the OC^High^ group, disease was controlled in 84.8%, 79.5%, and 61.5% of the patients with gastrointestinal, pancreatic, and other NETs, respectively (Figure 2C). In NET patients with high ^68^Ga‐DOTATATE avidity (SUVmax > 20), the DCRs were high in both groups, i.e., 97.7% and 91.4% in the OC^Low^ and OC^High^ groups, respectively (Figure 2D). Of note, in NET patients with low ^68^Ga‐DOTATATE avidity (SUVmax ≤ 20), DCR was 100% in the OC^Low^ group, which substantially decreased to 56.2% in the OC^High^ group (Figure 2D). DCR remained significantly better in the OC^Low^ group until 2.5 years from the commencement of PRRT, compared to the OC^High^ group (Figure 2E).

In NET patients in the OC^High^ group, higher grade tumors, high disease burden, or low ^68^Ga‐DOTATATE avidity were negative predictors of disease control on PRRT. To the contrary, NET patients in the OC^Low^ group achieved > 90% DCR even with higher tumor grade or disease burden, or low ^68^Ga‐DOTATATE avidity. Thus, RNA‐Seq deconvoluted baseline circulating OC% may be a reliable indicator of response to PRRT.

### 3.4 Evaluation of the Association Between Pre‐PRRT OC% With Progression Free Survival

The median follow‐up of NET patients for PFS was 25 months (IQR = 16–37) (Table 1). During the follow‐up period, disease progression was observed in 35 patients (25.55%) (Table 1). We assessed the prognostic potential of OC% in PRRT‐treated NET patients through Cox proportional hazards analyses. The median OC% in HDs (0.18) was selected as a cut‐off value, and the NET patients were categorized into OC^Low^ (≤ 0.18%) and OC^High^ (> 0.18%) groups.

In univariate analysis, tumor grade, disease burden, bone metastasis, ^68^Ga‐DOTATATE avidity, ^18^F‐FDG avidity, and higher OC% were significantly associated with PFS (Table 3). Age, sex, and liver metastases did not have a significant correlation with disease progression (Table 3). Patients with higher tumor grade had an increased risk of progression [G2 tumors: HR = 5.1, 95% CI = 1.53–16.98, *p* = 0.008; G3: HR = 21.04, 95% CI = 5.15–85.93, *p* < 0.0001 with G1 tumors as reference] (Table 3). Similarly, those with high disease burden [HR = 2.82, 95% CI = 1.43–5.55, *p* = 0.0027 with low disease burden as reference], bone metastases [HR = 3.87, 95% CI = 1.93–7.73, *p* = 0.00013 compared to patients with no bone metastasis], or patients with high ^18^F‐FDG avidity [HR = 2.1, 95% CI = 1.02–4.34, *p* = 0.044 compared to patients with low ^18^F‐FDG avidity] had significantly higher risk of disease progression (Table 3). Conversely, patients with high ^68^Ga‐DOTATATE avidity correlated with reduced hazard [HR = 0.35, 95% CI = 0.17–0.7, *p* = 0.003 compared to patients with low ^68^Ga‐DOTATATE avidity] (Table 3).

**TABLE 3:** Univariate and multivariate COXPH analyses for PFS.

Pre‐PRRT clinical characteristics	Univariate analysis		Multivariate analysis	
	HR (95% CI)	*p*	HR (95% CI)	*p*
Age: High (> 50 years)	1	1	1	1
Age: Low (≤ 50 years)	0.93 (0.48–1.81)	0.83	0.94 (0.45–1.95)	0.867
Sex: Female	1	1	1	1
Sex: Male	1.2 (0.6–2.42)	0.60	0.86 (0.38–1.92)	0.71
Tumor grade: G1	1	1	1	1
Tumor grade: G2	**5.1 (1.53–16.98)**	**0.008**	**4.44 (1.17–16.87)**	**0.029**
Tumor grade: G3	**21.04 (5.15–85.93)**	**0.00002**	**19.42 (4.16–90.59)**	**0.00016**
Low disease burden	1	1	1	1
High disease burden	**2.82 (1.43–5.55)**	**0.0027**	**2.79 (1.20–6.49)**	**0.017**
No liver metastasis	1	1	1	1
Liver metastasis	0.96 (0.43–2.11)	0.91	0.82 (0.35–1.91)	0.65
No bone metastasis	1	1	1	1
Bone metastasis	**3.87 (1.93–7.73)**	**0.00013**	**2.78 (1.13–6.8)**	**0.026**
^68^Ga‐DOTATATE avidity (≤ 20)	1	1	1	1
^68^Ga‐DOTATATE avidity (> 20)	**0.35 (0.17–0.7)**	**0.003**	**0.38 (0.17–0.88)**	**0.023**
^18^F‐FDG avidity (≤ 5)	1	1	1	1
^18^F‐FDG avidity (> 5)	**2.1 (1.02–4.34)**	**0.044**	1.08 (0.48–2.46)	0.85
OC% (≤ 0.18)	1	1	1	1
OC% (> 0.18)	**3.2 (1.32–7.75)**	**0.010**	**2.08 (0.78–5.54)**	**0.14**

*Note:* Bold values indicate significant associations (*p* < 0.05).

**FIGURE 3:**
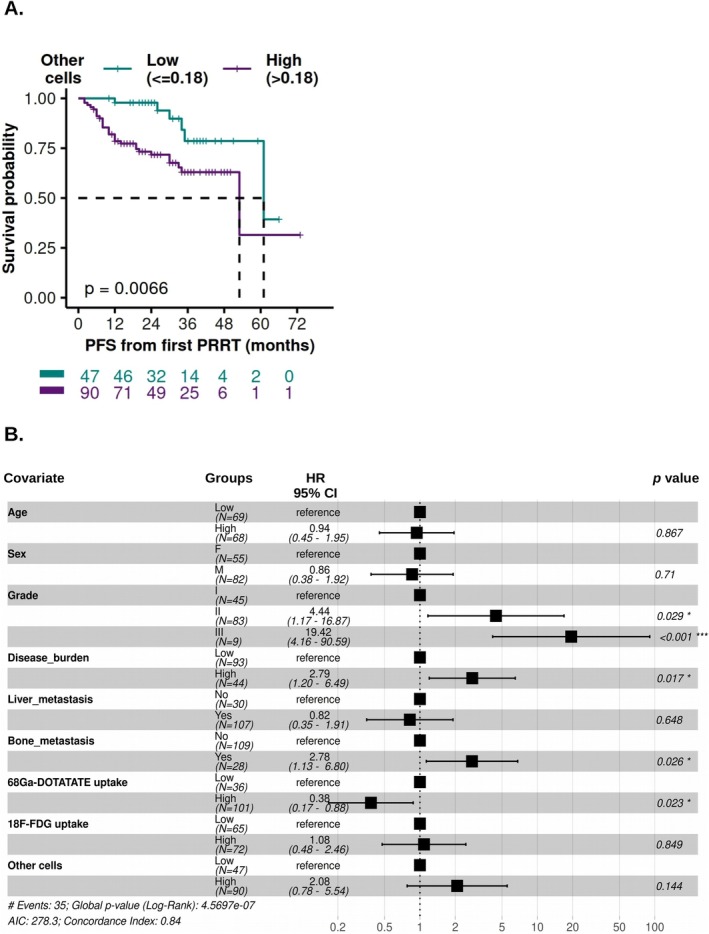
Association of pre‐PRRT OC% with PFS. (A) The Kaplan–Meier survival curve shows the correlation between pre‐PRRT OC% and progression‐free survival (PFS). In the survival curves, a cut‐off value of 0.18 was used to dichotomize NET patients into two groups: high cell proportions (> 0.18) and low cell proportions (≤ 0.18). Vertical drops in the curves indicate events (disease progression), while vertical marks represent right‐censored patients. Dashed lines indicate the median survival times. (B) Multivariate Cox proportional hazards (COXPH) analysis demonstrating the association of pre‐PRRT cell proportions with progression‐free survival (PFS). In the forest plot, hazard ratios (HRs) with 95% confidence intervals (CIs) and *p* values are shown. Significant associations with patient outcomes (*p* ≤ 0.05) are summarized with asterisks. *p ≤ 0.05, ***p ≤ 0.001.

Patients in the OC^High^ group (> 0.18%) had a significantly higher risk of early disease progression in univariate analysis [HR = 3.2, 95% CI = 1.32–7.75, *p* = 0.010] (Table 3). Kaplan–Meier plot demonstrated that patients in the OC^High^ group had significantly shorter progression‐free survival (53 months) compared to those with lower proportions (61 months, *p* = 0.0066) (Figure 3A). However, after adjusting for age, sex, tumor grade, disease burden, liver and bone metastasis, ^68^Ga‐DOTATATE and ^18^F‐FDG avidity, the association of OC% with PFS retained only marginal significance [HR = 2.08, 95% CI = 0.78–5.54, *p* = 0.14] in a multivariate model (Figure 3B and Table 3).

**FIGURE 4:**
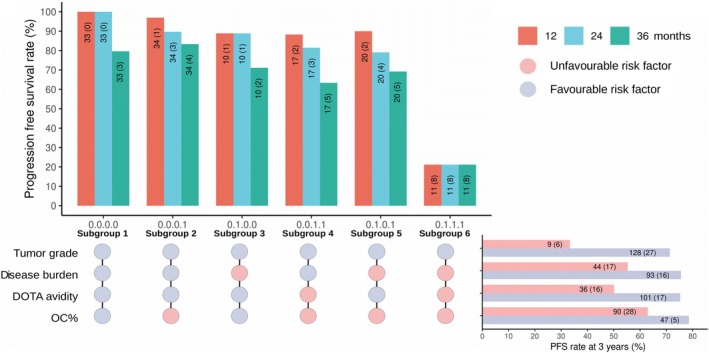
UpSet plot depicting the risk of disease progression in the subgroups defined by tumor grade, disease burden, ^68^Ga‐DOTATATE avidity, and OC%. Subgroups with ≥ 10 patients were included. The matrix panel below shows how subgroups were formed with favorable variables (G1/G2 tumor grade, low disease burden, high ^68^Ga‐DOTATATE avidity, or low OC%) indicated in blue and unfavorable variables (G3 tumor grade, high disease burden, low ^68^Ga‐DOTATATE avidity, or high OC%) in red. The bar plot at the top indicates the percentage of patients with progressive disease at 12 (red), 24 (blue) and 36 (green) months from the first PRRT cycle. The horizontal bar plot on the right depicts the effect of individual variables on disease progression, assessed at 3 years. The numbers of patients at risk at the start of PRRT are indicated, and the number of events (disease progression) is shown in parentheses.

Furthermore, we evaluated the risk of disease progression across subgroups defined by different combinations of favorable and/or unfavorable profiles of four key risk variables. The UpSet plot shows the proportions of patients with progressive disease across six subgroups (having ≥ 10 patients) at different time points after the first PRRT cycle. The observed risk of disease progression in the first 3 years of PRRT was the lowest in subgroup 1, which had all favorable risk factors (Figure 4). Subgroups 2–5 with one or two unfavorable variables showed increased risk of disease progression (Figure 4). Of note, subgroup 6—patients with well‐differentiated NETs, high disease burden, low ^68^Ga‐DOTATATE avidity (SUVmax: 15–20 in 8 patients and 10–15 in 3 patients), and high OC%—exhibited the highest risk of disease progression (top bar plot in Figure 4), an outcome not anticipated from the influence of (a) individual risk parameters in isolation (right bar plot in Figure 4), and (b) the binary combination of OC% with risk parameters (Figure 2). The risk of disease progression in Subgroup 6 is remarkably different compared to Subgroups 2–5 patients, who had only 1 or 2 unfavorable risk factors (top bar plot in Figure 4). Additionally, two G3 NET patients with low disease burden, high ^68^Ga‐DOTATATE avidity, and high OC%, had progressive disease within 12 months of starting PRRT (Data not shown). Thus, integrating OC% with established risk predictors has clinical value for stratifying NET patients for therapy, predicting response and disease progression.

### 3.5 Assessment of the Correlation Between Pre‐PRRT OC% and Overall Survival

**FIGURE 5:**
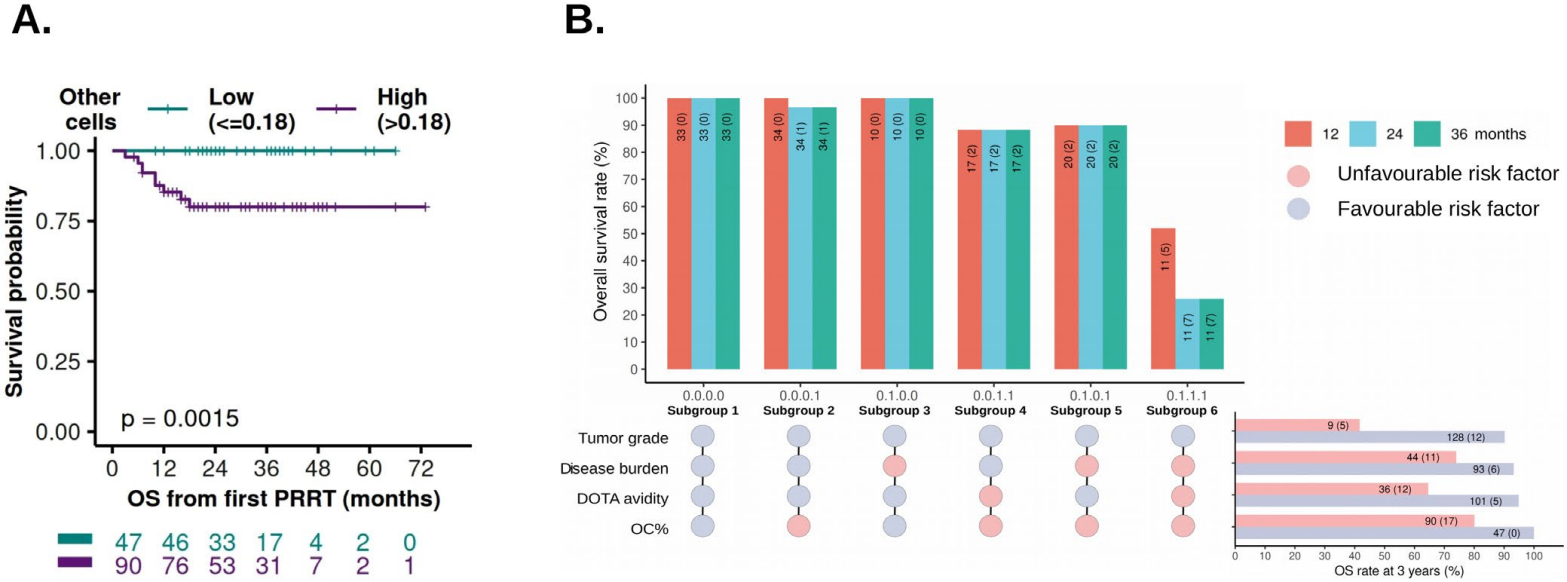
Association between overall survival, OC%, and other risk variants. (A) Kaplan–Meier survival curves illustrating the correlation between pre‐PRRT OC% and overall survival. Other details were the same as in Figure 3A. (B) UpSet plot depicting the differences in OS in different subgroups formed by combining 4 risk variables. Other details were the same as defined for Figure 4.

Overall survival remains a gold standard and a definitive, unbiased endpoint for demonstrating the efficacy of any cancer therapy [33]. In this study, the median follow‐up for OS was 26 months (IQR = 18–39) (Table 1). During the follow‐up period, 17 patients (11.77%) succumbed (Table 1). Of note, when the relationship between OC% and OS outcomes was evaluated, all recorded death events occurred in the OC^High^ group, indicating a significant negative correlation with OS (*p* = 0.0015) (Figure 5A). Median survival was not reached in either group. Additionally, univariate and Cox proportional hazards (CoxPH) analyses could not be conducted due to an indefinite hazard (all death events) in the OC^High^ group. The UpSet plot revealed remarkably adverse prognosis in subgroup 6 patients (7/11 died within 24 months of starting PRRT) (Figure 5B). Factually, the majority (10/11) of subgroup 6 patients were non‐responders (SD + PD according to RECIST 1.1 criteria). These findings support our hypothesis that OC%, along with established risk predictors, has clinical value in risk stratification and prognosis prediction in NET patients.

## 4 Discussion

In the past decade, PRRT with ^177^Lu‐DOTATATE has been established as a molecular therapy for somatostatin receptor (SSTR) expressing NETs, achieving an impressive disease control rate of approximately 80% [34, 35, 36, 37]. PRRT is preferred over alternative treatment modalities due to its tumor selectivity, minimal toxicity, and excellent tolerability in patients with advanced metastatic NETs [38]. Prognosis assessment in NET patients relies on the tumor‐node‐metastasis (TNM) classification, with disease stage considered a critical risk factor, alongside clinicopathologic characteristics such as age, sex, marital status, tumor size, tumor grade, and tumor site, all of which are significantly associated with survival [39, 40].

Molecular markers of prognosis are an emerging field of interest, offering direct utility for clinicians in stratifying patients for effective and cost‐efficient treatment regimens to improve outcomes. To that end, various liquid biopsy assays are either in use or under development, e.g., serum Chromogranin A (CgA) levels for assessment of tumor burden and disease progression [41], CTC counts for survival [16, 17, 18], multi‐gene diagnostic classifiers such as NETest, PRRT‐predictive‐quotient (PPQ), and NETseq have demonstrated high sensitivity in detecting NET disease and/or predicting response to PRRT [29, 42], and pathway‐based multi‐gene circulating biomarkers, heme metabolism and IL2/STAT5 signaling, show independent prognostic impact on PFS, in addition to tumor grade, disease burden and ^68^Ga‐DOTATATE avidity [9]. However, there is limited knowledge on the molecular determinants of prognosis in NETs. Thus, the current study explored a novel digital deconvolution metric—OC% to detect and quantify uncharacterized circulating cells from RNA‐Seq data and evaluated its utility as a predictor of PRRT efficacy and survival outcome.

Uncharacterized “other cells” circulating in the blood include CTCs, atypical immune cells, cancer stem cells, stromal cells, endothelial cells, or epithelial cells. While these cell types are rare in HDs, an increase in circulating endothelial cells has been documented in the presence of tissue injury, infection, or other underlying disease conditions [22]. Similarly, circulating epithelial or stromal cells are detectable in patients with benign bowel [43, 44] or liver diseases [45]. Consistent with these reports, we observed a baseline median OC% of 0.18 in HDs, which was significantly lower than the OC% in the NET patients (median = 0.26) (Figure 1A and Table 2).

In the NET patients studied, OC% showed a strong positive correlation with critical clinical covariates such as tumor grade and disease burden (Figure 1B,C and Table 2). These results corroborate earlier studies linking higher CTC counts with higher tumor burden and grade in midgut NETs [46]. Patients who progressed on PRRT had high OC% (median = 0.41) compared to those with controlled disease (median = 0.23) (Figure 1E and Table 2). Thus, pre‐PRRT OC% may serve as a surrogate marker for the efficacy of PRRT.

In binary analyses, NET patients in the OC^Low^ group achieved better (≥ 90%) than average DCR (~80%) on PRRT, despite having higher tumor burden (*n* = 10) or grade (G2 = 28, G3 = 1), or low ^68^Ga‐DOTATATE avidity (*n* = 4) (Figure 2). Tumor burden and grade are the known negative predictors of survival outcome [47]. This paradox, where patients with high tumor burden or grade but low OC% responding better to PRRT, may reflect differences in tumor biology, such as lower epithelial‐mesenchymal transition (EMT) and a more favorable immune microenvironment with fewer CTCs [48]. These findings underscore the potential of baseline OC% as a complementary biomarker alongside conventional clinical metrics for prognosis prediction.

On the contrary, DCR in the OC^High^ group showed a wide range (25%–96.3%), influenced by various tumor characteristics (Figure 2). Patients in the OC^High^ group with G1 or G2 tumors, low disease burden, gastrointestinal or pancreatic origin or high ^68^Ga‐DOTATATE avidity achieved standard DCR on PRRT or better (> 78%) (Figure 2). Of note, OC^High^ group patients with G3 tumors, low ^68^Ga‐DOTATATE avidity, other than gastroenteropancreatic primary site or high disease burden were poor responders to PRRT (Figure 2). NET patients in the OC^High^ group experienced significantly shorter PFS and OS in univariate analyses (Figures 3A and 5A, Table 3). However, in multivariate analysis, OC% retained only marginal significance for PFS [HR = 2.08, 95% CI = 0.78–5.54, *p* = 0.14] (Figure 3B, Table 3). This finding likely reflects the strong correlation between OC% and the established prognostic factors, including tumor grade (*p* = 0.029), disease burden (*p* = 0.016), and ^68^Ga‐DOTATATE avidity (*p* = 0.0026). It suggests that OC% captures critical biological and clinical information relevant to PRRT response, yet its prognostic value may be partially redundant with these parameters in the multivariate model. Therefore, we foresee that the OC% metric can be combined with the existing prognosis predictors (e.g., SSTR avidity, tumor grade, Chromogranin A, multi‐gene molecular assays) to enhance risk stratification tools or prognostic models.

To guide clinical decisions, we provided an at‐a‐glance color‐coded UpSet plot that integrates the combined effects of critical risk variables on prognosis in PRRT‐treated NET patients (Figures 4 and 5B). Here, we report a subgroup of NET patients—with well‐differentiated NETs, high disease burden, low ^68^Ga‐DOTATATE avidity, and high OC%—at a higher risk of adverse prognosis on PRRT. Thus, the pre‐PRRT OC% metric, when integrated with other risk factors, is valuable for clinicians in stratifying patients early on for a combination treatment regimen: “Sandwich” chemo‐PRRT [49], peptide receptor chemoradionuclide therapy [50], etc.

The OC% is a digitally deconvoluted RNA‐Seq‐derived metric. In this study, we demonstrated a significant improvement in risk stratification of patients by combining OC% with clinical covariates, e.g., tumor grade, disease burden, and SSTR avidity (Figures 4 and 5B). In the future, the OC% can be seamlessly integrated with assays that use RNA‐Seq technology (e.g., NETseq ensemble classifier [29], pathway‐based biomarkers [9]). For other liquid biopsy assays (e.g., ELISA‐based CgA test, flow cytometry‐derived CTC counts, or qPCR‐based NETest/PPQ multi‐gene assays), integrating OC% will require whole blood bulk RNA‐Seq through next generation sequencing (NGS). Nevertheless, the affordability and quick turnaround time of NGS (< $ 100/sample, ⁓1–2 days), and free availability and applicability of the Kassandra‐B deconvolution algorithm (https://science.bostongene.com/kassandra) for OC% estimation [27] will facilitate the development of multi‐analyte composite biomarkers for potential translation into clinical practice and integration into clinical decision workflows [51].

OC% could also serve as a prognostic biomarker in NET patients treated with other modalities, such as surgery, chemotherapy, or other targeted therapies, wherein disease burden, tumor grade and SSTR expression remain key determinants of prognosis [52]. Such efforts could establish a unified, robust, multi‐parametric prognostic model to guide patient selection for therapy and ultimately improve overall clinical outcomes for NET patients. This study presents a conceptual advance in the field of cancer prognostics. The molecularly‐derived OC% is an economical alternative to the cell enumeration methods and shows a high correlation with the most important prognosis predictors of NETs.

Though we report OC% as a promising predictive marker, a number of limitations to this study should be considered before generalizing the findings. This is a single‐center and retrospective study; thus, validation in a larger and preferably multi‐center NET patient cohort is warranted. We are currently recruiting patients for a prospective validation in our center. This study exclusively evaluated the significance of OC% in PRRT‐treated NET patients. The utility of this approach in NET patients receiving other therapeutic interventions needs further assessment. The Kassandra‐B algorithm deconvoluted other cells *in toto*. In future, more advanced algorithms deconvoluting individual “other cell” types may offer better prognostic value. Methodologies used for sample processing and transcriptome sequencing may influence the cut‐off value of OC%. Nevertheless, future prognostic classifiers may benefit from the integration of clinical and molecular prognosis predictors for evidence‐based risk stratification, treatment planning, and prognosis assessment in NET patients.

## Author Contributions

**Mahesh K. Padwal:** data curation (lead), formal analysis (lead), methodology (lead), software (lead), visualization (equal), writing – original draft (equal). **Rahul V. Parghane:** data curation (lead), investigation (equal), resources (equal). **Sandip Basu:** conceptualization (equal), data curation (supporting), investigation (equal), resources (equal), writing – review and editing (equal). **Bhakti Basu:** conceptualization (equal), project administration (lead), supervision (lead), visualization (equal), writing – original draft (equal), writing – review and editing (lead).

## Funding

The authors have nothing to report.

## Ethics Statement

The study was reviewed and approved by the Institutional Scientific Advisory Committee (SAC) and the Institutional Ethics Committee (IEC) at the Radiation Medicine Center, Bhabha Atomic Research Center (Approval No. P18/Feb/2019). The study was carried out as per the ethical tenets of the Helsinki Declaration.

## Consent

All participants signed written informed consent.

## Conflicts of Interest

The authors declare no conflicts of interest.

## Supporting information


**Appendix S1:** Clinical annotations of NET patients (NET1–NET137) and healthy donors (HD1–HD81).

## Data Availability

Data on OC% in all participants is provided in the Appendix S1. Clinical data analyzed in this study is available from the corresponding author upon reasonable request.

## References

[1] G. Klöppel , “Neuroendocrine Neoplasms: Dichotomy, Origin and Classifications,” Visceral Medicine 33 (2017): 324–330, 10.1159/000481390. 29177160 PMC5697503

[2] J. Y. Kim , S. M. Hong , and J. Y. Ro , “Recent Updates on Grading and Classification of Neuroendocrine Tumors,” Annals of Diagnostic Pathology 29 (2017): 11–16, 10.1016/j.anndiagpath.2017.04.005. 28807335

[3] W. H. M. Verbeek , C. M. Korse , and M. E. T. Tesselaar , “GEP‐NETs UPDATE: Secreting Gastro‐Enteropancreatic Neuroendocrine Tumours and Biomarkers,” European Journal of Endocrinology 174 (2016): R1–R7, 10.1530/eje-14-0971. 26162406

[4] S. Das and A. Dasari , “Epidemiology, Incidence, and Prevalence of Neuroendocrine Neoplasms: Are There Global Differences?,” Current Oncology Reports 23 (2021): 43, 10.1007/s11912-021-01029-7. 33719003 PMC8118193

[5] A. Ramesh , A. Chatterjee , and R. M. Subramaniam , “Neuroendocrine Neoplasms: Epidemiology, Diagnosis, and Management,” PET Clinics 18 (2023): 161–168, 10.1016/j.cpet.2022.10.002. 36707369

[6] I. M. Modlin , K. Oberg , D. C. Chung , et al., “Gastroenteropancreatic Neuroendocrine Tumours, the Lancet,” Oncology 9 (2008): 61–72, 10.1016/S1470-2045(07)70410-2. 18177818

[7] V. S. Jayaprakasam and L. Bodei , “Neuroendocrine Tumor Therapy Response Assessment,” PET Clinics 18 (2023): 267–286, 10.1016/j.cpet.2022.11.009. 36858748

[8] K. Sitani , R. V. Parghane , S. Talole , and S. Basu , “Long‐Term Outcome of Indigenous (177)Lu‐DOTATATE PRRT in Patients With Metastatic Advanced Neuroendocrine Tumours: A Single Institutional Observation in a Large Tertiary Care Setting,” British Journal of Radiology 94 (2021): 20201041, 10.1259/bjr.20201041. 33095671 PMC7774689

[9] M. K. Padwal , R. V. Parghane , A. Chakraborty , et al., “Systemic Cancer Hallmarks as Novel Markers Associated With Progression‐Free Survival in Gastroenteropancreatic Neuroendocrine Tumor Patients Undergoing Peptide Receptor Radionuclide Therapy,” Neuroendocrinology (2024): 1–14, 10.1159/000542918. 39626642

[10] M. K. Padwal , A. K. Nazar , R. V. Parghane , S. Basu , and B. Basu , “Evaluating the Prognostic Significance of the Pre‐Treatment Neutrophil‐to‐Lymphocyte and Monocyte‐to‐Lymphocyte Ratios in ^177^Lu‐DOTATATE PRRT Treated Patients With Advanced Metastatic Neuroendocrine Tumors,” Endocrine 89 (2025): 308–321, 10.1007/s12020-025-04212-z. 40131599 PMC12227452

[11] D. Lin , L. Shen , M. Luo , et al., “Circulating Tumor Cells: Biology and Clinical Significance,” Signal Transduction and Targeted Therapy 6 (2021): 404, 10.1038/s41392-021-00817-8. 34803167 PMC8606574

[12] A. W. Lambert , D. R. Pattabiraman , and R. A. Weinberg , “Emerging Biological Principles of Metastasis,” Cell 168 (2017): 670–691. 28187288 10.1016/j.cell.2016.11.037 PMC5308465

[13] R. Lawrence , M. Watters , C. R. Davies , K. Pantel , and Y.‐J. Lu , “Circulating Tumour Cells for Early Detection of Clinically Relevant Cancer,” Nature Reviews Clinical Oncology 20 (2023): 487–500, 10.1038/s41571-023-00781-y. PMC10237083 37268719

[14] C. Reduzzi , E. Nicolo’ , S. Singhal , et al., “Unveiling the Impact of Circulating Tumor Cells: Two Decades of Discovery and Clinical Advancements in Solid Tumors,” Critical Reviews in Oncology/Hematology 203 (2024): 104483, 10.1016/j.critrevonc.2024.104483. 39159706

[15] K. E. Sundling and A. C. Lowe , “Circulating Tumor Cells: Overview and Opportunities in Cytology,” Advances in Anatomic Pathology 26 (2019): 56–63, 10.1097/pap.0000000000000217. 30325755

[16] M. S. Khan , A. Kirkwood , T. Tsigani , et al., “Circulating Tumor Cells as Prognostic Markers in Neuroendocrine Tumors,” Journal of Clinical Oncology 31 (2012): 365–372, 10.1200/JCO.2012.44.2905. 23248251

[17] D. Mandair , M. S. Khan , A. Lopes , et al., “Prognostic Threshold for Circulating Tumor Cells in Patients With Pancreatic and Midgut Neuroendocrine Tumors,” Journal of Clinical Endocrinology & Metabolism 106 (2021): 872–882, 10.1210/clinem/dgaa822. 33180939

[18] J. C.‐H. Hsieh , G.‐Y. Chen , D. D.‐W. Jhou , et al., “The Prognostic Value of Circulating Tumor Cells in Asian Neuroendocrine Tumors,” Scientific Reports 9 (2019): 19917, 10.1038/s41598-019-56539-z. 31882775 PMC6934482

[19] M. Russano , A. Napolitano , G. Ribelli , et al., “Liquid Biopsy and Tumor Heterogeneity in Metastatic Solid Tumors: The Potentiality of Blood Samples,” Journal of Experimental & Clinical Cancer Research 39 (2020): 95, 10.1186/s13046-020-01601-2. 32460897 PMC7254767

[20] K. P. Gardner , M. Aldakkak , C.‐M. Tang , S. Tsai , and D. L. Adams , “Circulating Stromal Cells in Resectable Pancreatic Cancer Correlates to Pathological Stage and Predicts for Poor Clinical Outcomes,” npj Precision Oncology 5 (2021): 25, 10.1038/s41698-021-00161-8. 33742084 PMC7979885

[21] D. Adams , S. H. Lin , H. I. Pass , et al., “Circulating Stromal Cells as a Potential Blood‐Based Biomarker for Screening Invasive Solid Tumors,” Journal of Clinical Oncology 38 (2006): 3535.

[22] P. K. Goon , G. Y. Lip , C. J. Boos , P. S. Stonelake , and A. D. Blann , “Circulating Endothelial Cells, Endothelial Progenitor Cells, and Endothelial Microparticles in Cancer,” Neoplasia (New York, N.Y.) 8 (2006): 79–88, 10.1593/neo.05592. 16611400 PMC1578513

[23] F. Bertolini , Y. Shaked , P. Mancuso , and R. S. Kerbel , “The Multifaceted Circulating Endothelial Cell in Cancer: Towards Marker and Target Identification,” Nature Reviews Cancer 6 (2006): 835–845, 10.1038/nrc1971. 17036040

[24] H. J. Jeon , J. h. Seo , E. Jeong , et al., “Carcinoembryonic Antigen‐Positive Circulating Epithelial Cells as a Biomarker for the Diagnosis and Prognosis of Colorectal Cancer,” Biotechnology and Bioprocess Engineering 29 (2024): 877–889, 10.1007/s12257-024-00115-4.

[25] A. Cossarizza , H.‐D. Chang , A. Radbruch , et al., “Guidelines for the Use of Flow Cytometry and Cell Sorting in Immunological Studies (Third Edition),” European Journal of Immunology 51 (2021): 2708–3145, 10.1002/eji.202170126. 34910301 PMC11115438

[26] F. Avila Cobos , J. Alquicira‐Hernandez , J. E. Powell , P. Mestdagh , and K. De Preter , “Benchmarking of Cell Type Deconvolution Pipelines for Transcriptomics Data,” Nature Communications 11 (2020): 5650, 10.1038/s41467-020-19015-1. PMC7648640 33159064

[27] A. Zaitsev , M. Chelushkin , D. Dyikanov , et al., “Precise Reconstruction of the TME Using Bulk RNA‐Seq and a Machine Learning Algorithm Trained on Artificial Transcriptomes,” Cancer Cell 40 (2022): 879–894.e16, 10.1016/j.ccell.2022.07.006. 35944503

[28] E. A. Eisenhauer , P. Therasse , J. Bogaerts , et al., “New Response Evaluation Criteria in Solid Tumours: Revised RECIST Guideline (Version 1.1),” European Journal of Cancer 45 (2009): 228–247, 10.1016/j.ejca.2008.10.026. 19097774

[29] M. K. Padwal , R. V. Parghane , A. Chakraborty , et al., “Developing a Peripheral Blood RNA‐Seq Based NETseq Ensemble Classifier: A Potential Novel Tool for Non‐Invasive Detection and Treatment Response Assessment in Neuroendocrine Tumor Patients Receiving (177)Lu‐DOTATATE PRRT,” Journal of Neuroendocrinology 37 (2025): e13462, 10.1111/jne.13462. 39539072 PMC11919474

[30] N. L. Bray , H. Pimentel , P. Melsted , and L. Pachter , “Near‐Optimal Probabilistic RNA‐Seq Quantification,” Nature Biotechnology 34 (2016): 525–527, 10.1038/nbt.3519. 27043002

[31] R Core Team , R: A Language and Environment for Statistical Computing (2022), https://www.R‐project.org/.

[32] G. Rindi , O. Mete , S. Uccella , et al., “WHO Classification of Neuroendocrine Neoplasms,” Endocrine Pathology 33, no. 2022 (2022): 115–154, 10.1007/s12022-022-09708-2. 35294740

[33] S. H. Zhuang , L. Xiu , and Y. A. Elsayed , “Overall Survival: A Gold Standard in Search of a Surrogate: The Value of Progression‐Free Survival and Time to Progression as End Points of Drug Efficacy,” Cancer Journal (Sudbury, Mass.) 15 (2009): 395–400, 10.1097/PPO.0b013e3181be231d. 19826359

[34] L. F. Wang , L. Lin , M. J. Wang , and Y. Li , “The Therapeutic Efficacy of 177Lu‐DOTATATE/DOTATOC in Advanced Neuroendocrine Tumors: A Meta‐Analysis,” Medicine 99 (2020): e19304, 10.1097/md.0000000000019304. 32150065 PMC7478707

[35] J. Zhang , Q. Song , L. Cai , Y. Xie , and Y. Chen , “The Efficacy of (177)Lu‐DOTATATE Peptide Receptor Radionuclide Therapy (PRRT) in Patients With Metastatic Neuroendocrine Tumours: A Systematic Review and Meta‐Analysis,” Journal of Cancer Research and Clinical Oncology 146 (2020): 1533–1543, 10.1007/s00432-020-03181-2. 32281025 PMC11804348

[36] T. Brabander , W. A. van der Zwan , J. J. M. Teunissen , et al., “Long‐Term Efficacy, Survival, and Safety of [(177)Lu‐DOTA(0),Tyr(3)]octreotate in Patients With Gastroenteropancreatic and Bronchial Neuroendocrine Tumors,” Clinical Cancer Research: An Official Journal of the American Association for Cancer Research 23 (2017): 4617–4624, 10.1158/1078-0432.ccr-16-2743. 28428192

[37] J. R. Strosberg , M. E. Caplin , P. L. Kunz , et al., “(177)Lu‐Dotatate Plus Long‐Acting Octreotide Versus High‐Dose Long‐Acting Octreotide in Patients With Midgut Neuroendocrine Tumours (NETTER‐1): Final Overall Survival and Long‐Term Safety Results From an Open‐Label, Randomised, Controlled, Phase 3 Trial,” Lancet. Oncology 22 (2021): 1752–1763, 10.1016/s1470-2045(21)00572-6. 34793718

[38] S. Loharkar and S. Basu , “Peptide Receptor Radionuclide Therapy in Neuroendocrine Neoplasms and Related Tumors: From Fundamentals to Personalization and the Newer Experimental Approaches,” Expert Review of Precision Medicine and Drug Development 8 (2023): 1–32, 10.1080/23808993.2023.2211090.

[39] F. L. Greene and L. H. Sobin , “The Staging of Cancer: A Retrospective and Prospective Appraisal,” CA: A Cancer Journal for Clinicians 58 (2008): 180–190, 10.3322/ca.2008.0001. 18460593

[40] Z. Xu , L. Wang , S. Dai , et al., “Epidemiologic Trends of and Factors Associated With Overall Survival for Patients With Gastroenteropancreatic Neuroendocrine Tumors in the United States,” JAMA Network Open 4 (2021): e2124750, 10.1001/jamanetworkopen.2021.24750. 34554237 PMC8461504

[41] M. Franchina , F. Cavalcoli , O. Falco , M. La Milia , A. Elvevi , and S. Massironi , “Biochemical Markers for Neuroendocrine Tumors: Traditional Circulating Markers and Recent Development – A Comprehensive Review,” Diagnostics (Basel, Switzerland) 14 (2024): 1289, 10.3390/diagnostics14121289. 38928704 PMC11203125

[42] L. Bodei , N. Raj , R. K. Do , et al., “Interim Analysis of a Prospective Validation of 2 Blood‐Based Genomic Assessments (PPQ and NETest) to Determine the Clinical Efficacy of (177)Lu‐DOTATATE in Neuroendocrine Tumors,” Journal of Nuclear Medicine: Official Publication, Society of Nuclear Medicine 64 (2023): 567–573, 10.2967/jnumed.122.264363. 36396457 PMC10071782

[43] K. Pantel , E. Denève , D. Nocca , et al., “Circulating Epithelial Cells in Patients With Benign Colon Diseases,” Clinical Chemistry 58 (2012): 936–940, 10.1373/clinchem.2011.175570. 22205690

[44] A. M. Honan , G. E. Jacobsen , H. Drum , et al., “Stromal‐Like Cells Are Found in Peripheral Blood of Patients With Inflammatory Bowel Disease and Correlate With Immune Activation State,” Clinical and Translational Gastroenterology 15 (2024): e1, 10.14309/ctg.0000000000000721. PMC11421714 38829958

[45] I. Bhan , K. Mosesso , L. Goyal , et al., “Detection and Analysis of Circulating Epithelial Cells in Liquid Biopsies From Patients With Liver Disease,” Gastroenterology 155 (2018): 2016–2018.e11, 10.1053/j.gastro.2018.09.020. 30218669 PMC6347478

[46] T. Meyer , M. Caplin , M. S. Khan , et al., “Circulating Tumour Cells and Tumour Biomarkers in Functional Midgut Neuroendocrine Tumours,” Journal of Neuroendocrinology 34 (2022): e13096, 10.1111/jne.13096. 35132704 PMC9285714

[47] D. H. Dong , X. F. Zhang , A. G. Lopez‐Aguiar , et al., “Tumor Burden Score Predicts Tumor Recurrence of Non‐Functional Pancreatic Neuroendocrine Tumors After Curative Resection,” HPB: The Official Journal of the International Hepato Pancreato Biliary Association 22 (2020): 1149–1157, 10.1016/j.hpb.2019.11.009. 31822386 PMC10182413

[48] M. Yu , A. Bardia , B. S. Wittner , et al., “Circulating Breast Tumor Cells Exhibit Dynamic Changes in Epithelial and Mesenchymal Composition,” Science 339 (2013): 580–584, 10.1126/science.1228522. 23372014 PMC3760262

[49] R. V. Parghane , V. Ostwal , A. Ramaswamy , et al., “Long‐Term Outcome of “Sandwich” Chemo‐PRRT: A Novel Treatment Strategy for Metastatic Neuroendocrine Tumors With Both FDG‐ and SSTR‐Avid Aggressive Disease,” European Journal of Nuclear Medicine and Molecular Imaging 48 (2021): 913–923, 10.1007/s00259-020-05004-5. 32876706

[50] G. Kong , M. Thompson , M. Collins , et al., “Assessment of Predictors of Response and Long‐Term Survival of Patients With Neuroendocrine Tumour Treated With Peptide Receptor Chemoradionuclide Therapy (PRCRT),” European Journal of Nuclear Medicine and Molecular Imaging 41 (2014): 1831–1844, 10.1007/s00259-014-2788-5. 24844348 PMC4159597

[51] S. Basu , M. K. Padwal , R. V. Parghane , and B. Basu , “The Promise of Integrating Omics‐Driven Liquid Biopsy and Machine Learning Algorithms With Multi‐Tracer PET in the Management of Neuroendocrine Tumors: Envisioning a Panoptic Model for Precision Oncology and Molecular Theranostics,” European Journal of Nuclear Medicine and Molecular Imaging 52 (2025): 4761–4766, 10.1007/s00259-025-07398-6. 40481861

[52] R. Garcia‐Carbonero , J. Capdevila , G. Crespo‐Herrero , et al., “Incidence, Patterns of Care and Prognostic Factors for Outcome of Gastroenteropancreatic Neuroendocrine Tumors (GEP‐NETs): Results From the National Cancer Registry of Spain (RGETNE),” Annals of Oncology 21 (2010): 1794–1803, 10.1093/annonc/mdq022. 20139156

